# A link prediction approach to cancer drug sensitivity prediction

**DOI:** 10.1186/s12918-017-0463-8

**Published:** 2017-10-03

**Authors:** Turki Turki, Zhi Wei

**Affiliations:** 1 Department of Computer Science, King Abdulaziz University, P.O. Box 80221, Jeddah, 21589 Saudi Arabia; 2 Bioinformatics Program and Department of Computer Science, New Jersey Institute of Technology, Newark, NJ 07102 USA

**Keywords:** Link prediction, Feature learning, Precision medicine, Cancer drug discovery, Applications in biology and medicine

## Abstract

**Background:**

Predicting the response to a drug for cancer disease patients based on genomic information is an important problem in modern clinical oncology. This problem occurs in part because many available drug sensitivity prediction algorithms do not consider better quality cancer cell lines and the adoption of new feature representations; both lead to the accurate prediction of drug responses. By predicting accurate drug responses to cancer, oncologists gain a more complete understanding of the effective treatments for each patient, which is a core goal in precision medicine.

**Results:**

In this paper, we model cancer drug sensitivity as a link prediction, which is shown to be an effective technique. We evaluate our proposed link prediction algorithms and compare them with an existing drug sensitivity prediction approach based on clinical trial data. The experimental results based on the clinical trial data show the stability of our link prediction algorithms, which yield the highest *area under the ROC curve* (AUC) and are statistically significant.

**Conclusions:**

We propose a link prediction approach to obtain new feature representation. Compared with an existing approach, the results show that incorporating the new feature representation to the link prediction algorithms has significantly improved the performance.

**Electronic supplementary material:**

The online version of this article (doi:10.1186/s12918-017-0463-8) contains supplementary material, which is available to authorized users.

## Background

Cancer has a significant global impact on public health; it is the second leading cause of death in the United States of America [1]. Cancer patients respond differently to potential drugs (i.e., chemotherapy) due to environmental causes, tumor heterogeneity, and genetic factors, making cancer drug discovery difficult [2–5]. The increasing number of deaths associated with cancer has attracted the attention of researchers from numerous domains, such as computational biology, machine learning, and data mining [6–9]. Costello et al. [10] assessed the performance of 44 drug sensitivity prediction algorithms based on profiling datasets (i.e., genomic, proteomic, and epigenomic data) in breast cancer cell lines. The training set consists of 35 cell lines, in which each cell line is associated with 28 drug responses. The test set consists of 18 cell lines. The task of each prediction algorithm is to learn a model from the training cell lines and perform predictions on the test set. The predictions correspond to a ranking of the 28 drugs—from the most sensitive to the most resistant for each cell line on the test set. The top-performing approach [10] improved the performance by integrating several profiling datasets with improved representation with a probabilistic nonlinear regression model. The second-best performing approach employed random forest regression to make predictions on the test set. The prediction algorithms were evaluated using the weighted probabilistic *c*-index (wpc-index) and resampled Spearman correlations [10]. The remaining prediction algorithms were not statistically different.

Geeleher et al. [11] proposed the following approach to drug sensitivity in which the input data are baseline expressions with drug IC_50_ values in cell lines and in vivo tumor gene expressions. The raw microarray data for the cell lines and clinical trials are processed separately and then combined and homogenized. The homogenized expression data consist of cell line expression data (i.e., baseline gene expression levels in the cell lines) and clinical trial expression data (i.e., baseline tumor expression data from the clinical trial). A learning algorithm is applied to the cell line expression data with the associated drug IC_50_ values for cell lines to learn a model. The resulting model is applied to clinical trial expression data to yield drug sensitivity predictions.

Two problems associated with the previous drug sensitivity prediction algorithms contribute to the degradation of the performance: (1) the poor quality of cell lines, especially when cell lines are not screened against all compounds [12]; and (2) the failure to adopt a new feature representation, because new feature representations provide a basis for improving the performance of learning algorithms [13–15].

In this paper, we model the cancer drug sensitivity as a link prediction problem, which is a classical research topic in computational social science [16–19] and biomedicine [20, 21]. Modeling the problem as link prediction enables us to exploit two link prediction algorithms: (1) the supervised link prediction algorithm, which aims to select better quality cancer cell lines; and (2) the extended supervised link prediction, which selects cancer cell lines and the top-*k* genes (i.e., features) using state of the art CUR matrix decomposition [22]. Our experimental results indicate that the proposed link prediction algorithms outperform the baseline prediction algorithms proposed by Geeleher et al. [11].

The key contributions of our paper are as follows: 1) we represent cancer drug sensitivity as a link prediction problem, which to the best of our knowledge is the first robustly transfer cancer drug sensitivity prediction to link prediction, 2) we connect a social network domain to a health informatics domain for advancing health informatics, 3) we propose two link prediction algorithms, and 4) we perform an experimental study on clinical trial data to demonstrate the predictive power and stability of our proposed link prediction algorithms against the prediction algorithms that employ the current approach [11].

This paper is organized as follows: In Related works section, we review the relevant literature, which pertains to both link prediction and cancer drug sensitivity prediction. In Methods section, we describe how the cancer drug sensitivity problem can be modeled as a link prediction problem. Then, we propose two link prediction algorithms that employ our link prediction approach: the supervised link prediction algorithm (A1) and the extended supervised link prediction algorithm (A2). Results and experiments section reports the experimental results and compares our link prediction algorithms against the baseline on the clinical trial data that pertains to breast cancer and multiple myeloma. Conclusions section summarizes our contributions in this paper.

### Related works

#### Link prediction in gene regulatory networks

Given *m* genes, in which each gene has *n* expression values, we can denote their gene expression profiles by **G** ∈ **ℝ** ^*m* × *n*^, which contains *m* rows—each row corresponds to a gene—and *n* columns—each column corresponds to an expression value [23]. To learn a model, we need to know the regulatory relationships (i.e., labels) among the genes, which are stored in the matrix **H** ∈ **ℝ** ^*p* × 3^. **H** contains *p* rows—each row shows a known regulatory relationship between two genes—and three columns. The first column shows the source gene (i.e., the transcription factor). The second column shows the target gene, and the third column shows the label, which is denoted as +1 (i.e., present link) when the source gene regulates the target gene or −1 (i.e., missing link) when the source gene does not regulate the target gene. Thus, **H **represents the observed (i.e., known) gene regulatory network. To learn a model, we need to construct the training set** D** ∈ **ℝ** ^*p* × 2*n* + 1^. The *p* examples in **D** are constructed as follows: For each pair of genes with the associated label in matrix** H**, the *n* expression values of each pair of genes in matrix **G** are extracted, and the concatenation of the *n* expression values of each pair of genes and the corresponding label is performed. For example, consider the *i*th example in the training set **D** *,* which is denoted by **D**_*i*_ and defined as

1 $$ \kern0.5em {\mathbf{D}}_i=\left[{g}_i^{\mathsf{1}},{g}_i^{\mathsf{2}},\dots, {g}_i^n,{g}_l^{\mathsf{1}},{g}_l^{\mathsf{2}},\dots, {g}_l^n,{y}_i\right], $$

where ${g}_i^{\mathtt{1}},{g}_i^{\mathtt{2}},\dots, {g}_i^n$ are the *n* expression values of ${\mathtt{g}}_i$ (also called the expression profile of ${\mathtt{g}}_i\Big),$), ${g}_l^{\mathtt{1}},{g}_l^{\mathtt{2}},\dots, {g}_l^n$ are the *n* expression values of ${\mathtt{g}}_l$, and *y*_*i*_ ∈ {1, −1}. The *i*th example of the test set, **T**, is denoted by **T**_*i*_ and constructed as follows:

2 $$ \kern0.5em {\mathbf{T}}_i=\left[{g}_i^{\mathsf{1}},{g}_i^{\mathsf{2}},\dots, {g}_i^n,{g}_j^{\mathsf{1}},{g}_j^{\mathsf{2}},\dots, {g}_j^n\right], $$

where ${g}_i^{\mathtt{1}},{g}_i^{\mathtt{2}},\dots, {g}_i^n$ are the *n* expression values of ${\mathtt{g}}_i$, and ${g}_j^{\mathtt{1}},{g}_j^{\mathtt{2}},\dots, {g}_j^n$ are the *n* expression values of ${\mathtt{g}}_j$. These feature vector definitions have been used by the existing supervised inference of gene regulatory networks [23–28]. After constructing the feature vectors, the learning algorithm is applied to **D** to induce (i.e., learn) the model *h*. The resulting model is used to perform prediction on** T**. The known regulations among genes enable using the induction principle to predict new regulations (i.e., labels): If gene ${\mathtt{g}}_j$ has an expression profile that is similar to gene ${\mathtt{g}}_l$, which is known to be regulated by ${\mathtt{g}}_i$, then ${\mathtt{g}}_j$ is likely to be regulated by ${\mathtt{g}}_i$ [29]. Genes with similar expression profiles that are likely to be co-regulated have been used in the unsupervised clustering of expression profiles [30–32].

#### Cancer drug sensitivity prediction

The gene expression profiles denoted by X ∈ **ℝ** ^*p* × *n*^, which contains *p* rows—each row corresponds to a cell line or a sample—and *n* columns—each column corresponds to a gene. Y = (*y*_1_,  … , *y*_*p*_)^*T*^ consists of the corresponding real-value drug responses (i.e., drug IC_50_ values) to X, where Y ∈ **ℝ** ^*p*^ (i.e., the *p-*dimensional column vector). IC_50_ is defined as the concentration of a compound that is required to produce 50% cancer cell growth inhibition after 48 h of treatment [33]. A training set is defined as $\mathrm{D}={\left\{\left({\mathtt{g}}_i,{y}_i\right)\right\}}_{i=1}^p$, where ${\mathtt{g}}_i\in \mathrm{X}\;\mathrm{and}\kern0.24em {y}_i\in \mathrm{Y}.$. Let the *i*th example of the training set **D**, denoted by **D**_*i*_, be defined as

3 $$ \kern0.5em {\mathbf{D}}_{\boldsymbol{i}}=\left[{g}_i^{\mathsf{1}},{g}_i^{\mathsf{2}},\dots, {g}_i^n,{y}_i\right], $$

where ${g}_i^{\mathtt{1}},{g}_i^{\mathtt{2}},\dots, {g}_i^n$ represent the *n* genes of the cancer cell line ${\mathtt{g}}_i$ (also called the expression profile of ${\mathtt{g}}_i\Big),$), and *y*_*i*_ ∈ **ℝ** is the drug response value. The *i*th example of the test set **T**, denoted by **T**_*i*_, is constructed as follows:

4 $$ \kern0.5em {\mathbf{T}}_i=\left[{g}_j^{\mathsf{1}},{g}_j^{\mathsf{2}},\dots, {g}_j^n\right]. $$

These feature vector definitions have been used by existing supervised cancer drug sensitivity prediction algorithms [9–11, 33–36]. A learning algorithm is applied to **D** to induce model *h*, which is subsequently used to perform predictions on **T**. Known cancer cell lines with associated drug responses enabled the use of the induction principle: If tumor ${\mathtt{g}}_j$ has an expression profile similar to ${\mathtt{g}}_i$, then ${\mathtt{g}}_j$ is likely to have a drug response value closer to the drug response value associated with ${\mathtt{g}}_i$.

## Methods

The fundamental task of cancer drug sensitivity prediction is to correctly predict the response of a tumor to the drug. This prediction is typically achieved based on *how closely* this tumor (also referred to as the test example) is related to a known cancer cell line with the associated drug response. Proximity, which is a measure of closeness, lies at the heart of both link prediction in gene regulatory networks and cancer drug sensitivity prediction [29, 37].

### Feature vector construction

To bridge link prediction and cancer drug sensitivity, we transform the feature representations of Eqs. (3) and (4) to the corresponding Eqs. (1) and (2) as follows: Let ${\left\{\left({\mathtt{g}}_i,{y}_i\right)\right\}}_{i=1}^p\subseteq \mathbf{D}$ be the cancer cell lines, where** D** ∈ **ℝ** ^*p* × *n* + 1^ , *b* = *p*.

1. Find the *k* ^*’*^ nearest neighbors ${\mathtt{g}}_1^{\ast },{\mathtt{g}}_2^{\ast },\dots, {\mathtt{g}}_{k^{\hbox{'}}}^{\ast }$ of each ${\mathtt{g}}_i$ in **D**. (In this study *k* ^*’*^ *=* 1.)
2. Generate synthetic cell lines along the lines between the randomly selected *k* ^*’*^ nearest neighbors and each ${\mathtt{g}}_i$ using the following lines of code:
  2.1 for *i =* 1 to *p*
    2.1.1 for *j =* 1 to *k* ^*’*^
      2.1.1.1 *b* = *b* + 1
      2.1.1.2 $$ {\mathtt{g}}_b={\mathtt{g}}_i+\left({\mathtt{g}}_j^{\ast }-{\mathtt{g}}_i\right)\lambda $$
      2.1.1.3 Store $$ \left[{\mathtt{g}}_i,{\mathtt{g}}_b,{y}_i\right] $$ in **G**
    2.1.2 end for
  2.2 end for

where the index *b* refers to only those synthetic cell lines (e.g., ${\mathtt{g}}_{p+1}$ when the index *b* = *p* + 1) that differ from the cell lines in **D**, whose indexes run from 1 to *p*, *λ* = 0.3, and **G** ∈ **ℝ** ^*p* × 2*n* + 1^ is the new feature representation of the cell lines of the training set. Step 2.1.1.2 creates the synthetic cell line ${\mathtt{g}}_b$. Let **G**_*i*_ be the *i*th row of **G**, defined as

5 $$ {\mathbf{G}}_i=\left[{\mathrm{g}}_{\mathrm{i}}^{\mathsf{1}},{\mathrm{g}}_{\mathrm{i}}^{\mathsf{2}},\dots, {\mathrm{g}}_{\mathrm{i}}^{\mathrm{n}},{\mathrm{g}}_{\mathrm{p}+1}^{\mathsf{1}},{\mathrm{g}}_{\mathrm{p}+1}^{\mathsf{2}},\dots, {\mathrm{g}}_{\mathrm{p}+1}^{\mathrm{n}},{\mathrm{y}}_{\mathrm{i}}\right], $$

where ${\mathtt{g}}_i^{\mathtt{1}},{\mathtt{g}}_i^{\mathtt{2}},\dots, {\mathtt{g}}_i^n$ represent *n* genes of the cancer cell line ${\mathtt{g}}_i$, ${g}_{p+1}^{\mathtt{1}},{g}_{p+1}^{\mathtt{2}},\dots, {g}_{p+1}^n$ represent the synthetic *n* genes of the synthetic cancer cell line ${\mathtt{g}}_{p+1}$, and *y*_*i*_ ∈ **ℝ** denotes that both ${\mathtt{g}}_i$ and ${\mathtt{g}}_{p+1}$ are linked by sharing the same drug response value. Let ${\left\{\left({\mathtt{g}}_i,{y}_i\right)\right\}}_{i=1}^q\subseteq \mathbf{T}$ be the test set of tumors, where **T** ∈ **ℝ** ^*q* × *n*^. Note that Steps 1–2 are similar to the Synthetic Minority Oversampling Approach (SMOTE) [38, 39], However, Step 2.1.1.3 is a different core step in which we increase the dimensionality (i.e., the number of features) instead of the size, as SMOTE does. We then apply the previous steps (i.e., Steps 1 and 2—changing Step 2.1 to *i =* 1 to *q* and Step 2.1.1.3 to Store $\left[{\mathtt{g}}_i,{\mathtt{g}}_b\right]$ in **G** ^'^) to **T** to obtain **G** ^'^ ∈ **ℝ** ^*q* × 2*n*^. **G** ^'^ is the new feature representation of the clinical trial expression data of the test set. Let ${\mathbf{G}}_i^{\hbox{'}}$ be the *i*th row of **G** ^'^, which is defined as

6 $$ \kern0.5em {\mathbf{G}}_i^{\prime }=\left[{g}_j^{\mathsf{1}},{g}_j^{\mathsf{2}},\dots, {g}_j^n,{g}_{p+2{k}^{\prime }+1}^{\mathsf{1}},{g}_{p+2{k}^{\prime }+1}^{\mathsf{2}},\dots, {g}_{p+2{k}^{\prime }+1}^n\right]. $$

where ${\mathtt{g}}_j^{\mathtt{1}},{\mathtt{g}}_j^{\mathtt{2}},\dots, {\mathtt{g}}_j^n$ represent *n* genes of tumor ${\mathtt{g}}_j$, and ${\mathtt{g}}_{p+2{k}^{\hbox{'}}+1}^{\mathtt{1}},{\mathtt{g}}_{p+2{k}^{\hbox{'}}+1}^{\mathtt{2}},\dots, {\mathtt{g}}_{p+2{k}^{\hbox{'}}+1}^n$represent *n* synthetic genes of the synthetic tumor ${\mathtt{g}}_{p+2{k}^{\hbox{'}}+1}$. A learning algorithm is called on the training set, **G** to induce the model *h*, which is subsequently used to perform predictions on the test set **G** ^'^. The logic behind the mechanism of the induction principle is as follows: If the expression profiles of the pair of tumors $\left({\mathtt{g}}_j,{\mathtt{g}}_{p+2{k}^{\hbox{'}}+1}\right)$ are similar to those of the cell lines $\left({\mathtt{g}}_i,{\mathtt{g}}_{p+1}\right)$, then $\left({\mathtt{g}}_j,{\mathtt{g}}_{p+2{k}^{\hbox{'}}+1}\right)$ is likely to have a drug response value closer to the drug response value associated with $\left({\mathtt{g}}_i,{\mathtt{g}}_{p+1}\right)$. In machine learning terms, let $\left({\mathtt{g}}_i,{\mathtt{g}}_{p+1},{y}_i\right)\in {\mathbf{\mathbb{R}}}^{2n+1}$ be a row feature vector that encodes information about the pair of cancer cell lines $\left({\mathtt{g}}_i,{\mathtt{g}}_{p+1}\right)$. Given a new pair of tumors encoded by $\left({\mathtt{g}}_j,{\mathtt{g}}_{p+2{k}^{\hbox{'}}+1}\right)$, if $\left({\mathtt{g}}_j,{\mathtt{g}}_{p+2{k}^{\hbox{'}}+1}\right)$ has feature values similar to $\left({\mathtt{g}}_i,{\mathtt{g}}_{p+1}\right)$, whose label is* y*_*i*_, then $\left({\mathtt{g}}_j,{\mathtt{g}}_{p+2{k}^{\hbox{'}}+1}\right)$ is more likely to have a closer response (i.e., label) value to *y*_*i*_.

### Notations and algorithms

#### Notations

To provide a better understanding of our proposed prediction algorithms, the notations used throughout the remainder of this paper are summarized as follows: Matrices are denoted by boldface uppercase letters, e.g., matrix **X**. We denote the row vectors of a matrix by boldface uppercase letters with a subscript, e.g., **X**_*j*_ is the *j*th row of matrix** X**. Vectors are denoted by boldface lowercase letters, e.g., vector **v**. Vector entries are denoted by italic lowercase letters with a subscript, e.g., *v*_*i*_ is the *i*th entry of vector **v**. The number of entries of a vector is denoted by the cardinality symbol, e.g. ∣**v**∣ is the number of elements of vector **v**. Scalars are denoted by italic lowercase letters, e.g., *m. f* , *f* ^∗^ , and *h* are reserved letters, where *f* refers to a learning algorithm (e.g., SVR), *f* ^∗^ refers to an induced (i.e., learned) model, and *h* is an induced model used to perform predictions on the test set. We refer to specific learning algorithms and induced models using subscripts. For example, ${f}_i\left({f}_i^{\ast },\mathrm{respectively}\right)$ denotes the *i*th learning algorithm and induced model, respectively.

#### The supervised link prediction algorithm (A1)

**Fig. 1:**
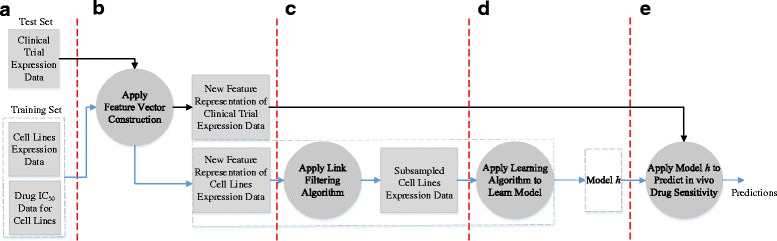
Data flow diagram that shows our supervised link prediction algorithm to predict in vivo drug sensitivity. (**a**) The training and test data are provided to the supervised link prediction algorithm. (**b**) A feature vector construction method is applied to the training and test data, to obtain new feature representations of the training and test data. (**c**) A link filtering algorithm is applied to the new feature representation of the training data, to yield subsampled data. (**d**) A learning algorithm takes as input the subsampled data, to induce the model *h*. (**e**) The model *h* is applied to the new feature representation of the test data, to yield predictions

Figure 1 outlines the supervised link prediction algorithm, which we designate A1, as follows. (a) Given a training set of cancer cell lines with associated drug responses **D** ∈ **ℝ** ^*p* × *n* + 1^ and a test set of tumors **T** ∈ **ℝ** ^*q* × *n*^ that are described as in cancer drug sensitivity prediction subsection. (b) Transform **D** and **T** using the feature vector construction method described in feature vector construction subsection, to obtain a new feature representation **G** ∈ **ℝ** ^*p* × 2*n* + 1^ for the training set and a new feature representation **G** ^'^ ∈ **ℝ** ^*q* × 2*n*^ for the test set. (c) Our link filtering method aims to select a better quality training set that works as follows: Each row (i.e., feature vector) in the new representations **G** and **G** ^'^ can be viewed as a cell line or tumor, represented by a 2*n*–dimensional row vector when the drug responses of the training set **G** are excluded. We weigh each cell line [40] ${\mathtt{g}}_i$ in the training set **G** by the minimum distance from the cell line ${\mathtt{g}}_i$ to all tumors ${\mathtt{g}}_j^{\hbox{'}}$ in the testing set **G** ^'^:

7 $$ \kern0.5em {w}_i=\mathrm{dist}\left({\mathbf{g}}_{\boldsymbol{i}},{\mathbf{g}}_{{\boldsymbol{j}}^{\ast}}^{\prime}\right)\ \mathbf{with}\kern0.75em {\boldsymbol{j}}^{\ast}=\boldsymbol{\arg}\underset{j\in \left\{1,\dots, q\right\}}{\mathit{\min}}\mathrm{dist}\left({\mathbf{g}}_{\boldsymbol{i}},{\mathbf{g}}_{\boldsymbol{j}}^{\prime}\right), $$

where ${\mathtt{g}}_i\in {\mathbf{\mathbb{R}}}^{2n}$, ${\mathtt{g}}_j^{\hbox{'}}\in {\mathbf{\mathbb{R}}}^{2n}$, *w*_*i*_ is the weight assigned to ${\mathtt{g}}_i$, and $\mathrm{dist}\left({\mathtt{g}}_i,{\mathtt{g}}_{j^{\ast}}^{\hbox{'}}\right)$ is the Euclidean distance. Let w = (*w*_1_, *w*_2_,  … , *w*_*p*_). Then, we perform the following steps to select better quality training cell lines using our modified version of *Query by Committee* (QBC) [41–43]:

1. Let *med* be the median of the w vector of weights of each ${\mathtt{g}}_i$ in **G**
2. Let $$ \mathbf{X}=\left\{\left({\mathtt{g}}_i,{y}_i\right)|\left({\mathtt{g}}_i,{y}_i\right)\in \mathbf{G}\;\mathrm{and}\;{w}_i\le med\right\} $$
3. Let $$ {\mathbf{X}}^{\hbox{'}}=\left\{{\mathtt{g}}_i|{\mathtt{g}}_i\kern0.24em \mathrm{in}\kern0.24em \mathbf{G}\;\mathrm{and}\;{w}_i\le med\right\} $$
4. Let $$ \mathbf{Z}=\left\{\left({\mathtt{g}}_i,{y}_i\right)|\left({\mathtt{g}}_i,{y}_i\right)\in \mathbf{G}\;\mathrm{and}\;{w}_i\ge med\right\} $$
5. Let $$ {\mathbf{Z}}^{\hbox{'}}=\left\{{\mathtt{g}}_i|{\mathtt{g}}_i\kern0.24em \mathrm{in}\kern0.24em \mathbf{G}\;\mathrm{and}\;{w}_i\ge med\right\} $$
6. Apply the learning algorithm, *f*_1_ or *f*_2_, to **X** or **Z**, respectively, to induce the model ${f}_1^{\ast}\;\left({f}_2^{\ast },\mathrm{respectively}\right)$. (In this study, we chose ridge regression as the learning algorithm)
7. Apply the model ${f}_1^{\ast}\;\left({f}_2^{\ast },\mathrm{respectively}\right)$ to perform predictions on **Z** ^'^ or **X**′, respectively) and store predictions in **v** or **b** respectively)
8. Let *q =* ∣**v**∣ = ∣**b**∣
9. Let **P** = (**v**, **b**)^*T*^
10.  Let **r** = {*y*_*i*_| *y*_*i*_ in **Z**} and  **e** = {*y*_*i*_| *y*_*i*_ in **X**}
11.  Let **R** = (**r**, **e**)^*T*^
12.  
j* $$ =\underset{j\in \left\{1,2\right\}}{\arg\;\max}\kern0.24em \frac{1}{q}{\left({\mathbf{P}}_j-{\mathbf{R}}_j\right)}^2 $$
13. $$ \mathbf{S}=\left\{\begin{array}{l}\mathbf{X}\kern0.36em \mathrm{if}\ {j}^{\ast }=1\\ {}\mathbf{Z}\kern0.24em \mathrm{otherwise}\end{array}\right. $$
14. $$ \mathbf{U}=\left\{\begin{array}{l}\mathbf{Z}\kern0.36em \mathrm{if}\ {j}^{\ast }=1\\ {}\mathbf{X}\kern0.24em \mathrm{otherwise}\end{array}\right. $$

QBC aims to partition the training set **G** into **S** and **U**, where **S** or **U **is treated as the labeled or unlabeled set, respectively. QBC is accompanied by two major items: (1) the set of models (i.e., the committee) that are consistent with all labeled cell lines in **S**; and (2) given the unlabeled set, **U**, the QBC applies the models (i.e., the committee) to **U** to select the unlabeled tumor that maximizes the disagreement because it represents the most important tumor that will be added to **S**, in addition to querying the drug response value associated with the tumor. The main obstacle of the first major step of QBC is to find models that agree on all the labels of set **S** with reasonable computational complexity [43]. Thus, we relax the first major step according to Steps 1–14, where relaxation is practiced to address the first major step [41]. Steps 1–5 partition the training set into **X** and **Z** using the median as a threshold, where **X** or **Z** contains cell lines from **G** that are near or far, respectively, from the test set **G** ^'^. Steps 6–14 aim to assign the set of cell lines where the model incurred fewer errors (or more errors, respectively) to **S** or **U**, respectively. The logic behind these steps (i.e., Steps 13–14) is that we want **S** or **U**, respectively, to contain the set of cell lines that are more or less, respectively, correctly labeled by one model (i.e., one member of the committee). Steps 1–14 are motivated by other QBC approaches [41–43], in which the success of the second major step of QBC is dependent on the first major step.

15 Repeat *k”* times
  15.1 Apply the learning algorithms *f*_1_ , *f*_2_ ,  …  , *f*_*t*_ on** S** to induce the models (i.e., committee) ${f}_1^{\ast },{f}_2^{\ast },\dots, {f}_t^{\ast }$. (In this study, *t =* 3, and the learning algorithms include support vector regression with a linear kernel (SVR + L), SVR with a polynomial kernel of degree 5, and SVR with a sigmoid kernel (SVR + S))
  15.2 Let ${w}_t^{\hbox{'}}$ be the weight of the *i*th model ${f}_i^{\ast }$ where ${\boldsymbol{w}}^{\hbox{'}}=\sum_{i=1}^t{w}_i^{\hbox{'}}=1$. (In this study, *t =* 3 and ${w}_1^{\hbox{'}}={w}_2^{\hbox{'}}={w}_3^{\hbox{'}}=\frac{1}{3}$)
  15.3 For each ${\mathtt{g}}_j$ in **U**, let ${f}^{\hbox{'}}\left({\mathtt{g}}_j\right)=\sum_{i=1}^t{\boldsymbol{w}}_i^{\hbox{'}}{f}_i^{\ast}\left({\mathtt{g}}_j\right)$ where ${f}_i^{\ast}\left({\mathtt{g}}_j\right)$ is the prediction of the *i*th learned model on the *j*th cell line ${\mathtt{g}}_j$, and ${f}^{\hbox{'}}\left({\mathtt{g}}_j\right)$ is the weighted ensemble average of the *j*th cell line ${\mathtt{g}}_j$.
  15.4 Find the cell line ${\mathtt{g}}_{j^{\ast }}$ that maximizes the disagreement:
    15.4.1. j* $$ =\underset{j\in \left\{1,\dots, |\mathbf{v}|\right\}}{\arg\;\max}\kern0.24em \sum_{i=1}^t{\boldsymbol{w}}_i^{\hbox{'}}{\left({f}_i^{\ast}\left({\mathtt{g}}_j\right)-{f}^{\hbox{'}}\left({\mathtt{g}}_j\right)\right)}^2 $$
  15.5 Find the label ${y}_{j^{\ast }}$ of ${\mathtt{g}}_{j^{\ast }}$ in **U**
  15.6 Add the pair $\left({\mathtt{g}}_{j^{\ast }},{y}_{j^{\ast }}\right)\in \mathbf{U}$ to **S** and remove the pair $\left({\mathtt{g}}_{j^{\ast }},{y}_{j^{\ast }}\right)$ from **U**
  15.7 Update ∣v∣ = ∣v∣ − 1
16 Return **S**

Steps 15.1–15.4.1 return the index of the cell line in set **U** that maximizes the disagreement, where disagreement is defined in Step 15.4.1 [44]. Then, $\left({\mathtt{g}}_{j^{\ast }},{y}_{j^{\ast }}\right)$ is added to or removed from **S** or **U** respectively, as shown in Steps 15.5–15.6. (In this study, *k” = 5*.) Step 15.7 updates |v| as the size of U is reduced after each iteration.** S** (Step 16) is the returned set that will be used as the training set. (d) We apply a learning algorithm on **S** to induce the model *h*. Finally (i.e., (e in Fig. 1)), we apply model *h* to perform predictions on the test set **G** ^'^ (i.e., the set of new feature representations of the clinical trial expression data). In the remainder of this paper, we refer to the supervised link prediction algorithms that employ the following machine learning algorithms (SVR and RR) as: A1 + SVR + L, A1 + SVR + S, and A1 + RR (abbreviations are listed in Table 1).

**Table 1:** Abbreviations of the drug sensitivity prediction algorithms

Abbreviation	Prediction Algorithm
A1 + SVR + L	The supervised link prediction algorithm using support vector regression with a linear kernel
A1 + SVR + S	The supervised link prediction algorithm using support vector regression with a sigmoid kernel
A1 + RR	The supervised link prediction algorithm using ridge regression
A2 + SVR + L	The extended supervised link prediction algorithm using support vector regression with a linear kernel
A2 + SVR + S	The extended supervised link prediction algorithm using support vector regression with a sigmoid kernel
A2 + RR	The extended supervised link prediction algorithm using ridge regression
B + SVR + L	The baseline approach using support vector regression with a linear kernel
B + SVR + S	The baseline approach using support vector regression with a sigmoid kernel
B + RR	The baseline approach using ridge regression

#### The extended supervised link prediction algorithm (A2)

**Fig. 2:**
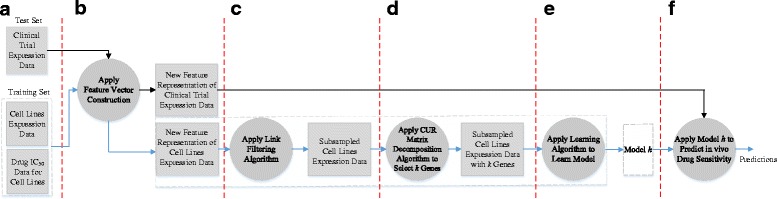
Data flow diagram showing the major steps in our extended supervised link prediction algorithm to predict in vivo drug sensitivity. (**a**) The training and test data are provided to the extended supervised link prediction algorithm. (**b**) A feature vector construction method is applied to the training and test data, to obtain new feature representations of the training and test data. (**c**) A link filtering algorithm is applied to the new feature representation of the training data, to yield subsampled data. (**d**) A feature selection step is applied to subsampled data, to obtain subsampled data with fewer features (i.e., genes). (**e**) A learning algorithm takes as input the subsampled data with fewer features, to induce the model *h*. (**f**) The features in the test data are selected using the same positions as in the training data and the model *h* is applied to the test data with the selected features, to yield predictions

Figure 2 shows the data flow diagram of the extended supervised link prediction (A2). Steps (a), (b), and (c) are the same as Steps (a), (b), and (c) of the supervised link prediction algorithm. (d) Mahoney et al. [22] proposed CUR matrix decomposition as a dimensionality reduction paradigm that aims to obtain a low rank approximation of matrix **S**, which is expressed in terms of the actual rows and columns of the original matrix **S**:

8 $$ \kern0.5em \mathbf{S}\approx \mathbf{CUR}, $$

where **C** consists of a small number of the actual columns of **S** *,* **R** consists of a small number of the actual rows, and **U** is a constructed matrix that guarantees that **CUR** is close to **S** *.* We select *k* genes based on their importance score (refer to Equation 9), which depends on matrix **S** and the input rank parameter *l* (in this study, we used the default parameter value for *l* in CUR function [45].) If ${v}_j^{\xi }$ is the *j*-th element of the *ξ* − th right singular vector of **S** *,* then the normalized statistical leverage scores are equal to

9 $$ \kern0.5em {\pi}_j=\frac{1}{l}\sum_{\xi =1}^l{\left({v}_j^{\xi}\right)}^2 $$

for all *j* = 1..2n, and $\sum_{j=1}^{2n}{\pi}_j=1$. Statistical leverage scores have been successfully employed in data analysis to identify the most influential genes and outlier detection [22]. A high statistical leverage score for a given gene indicates that the gene is regarded as an important (i.e., influential) gene. A low statistical leverage score for a given gene indicates that the gene is regarded as a less important gene. We store the indexes of the highest *k* leverage scores in **I**; these correspond to the positions of the *k* most influential genes in matrix **S** *.* We select *k* genes from the training set **S** using their positions in **I** and store subsampled cell line expression data with *k* genes in **S** ^'^. (e) A learning algorithm is called on **S** ^'^ to induce model *h*. (f) The *k* genes in the test set **G** ^'^ are selected using their positions in **I** and stored in **G** ^''^. Model *h* is applied on the test set **G** ^''^ to perform predictions. We refer to the extended supervised link prediction algorithms that employ machine learning algorithms as A2 + SVR + L, A2 + SVR + S, and A2 + RR (see Table 1).

## Results

We empirically evaluate our proposed approach and compare it against the baseline approach proposed by Geeleher et al. [11] on clinical trial datasets. This section first describes the datasets and experimental methodology and presents the experimental results.

### Datasets

#### Data pertaining to breast cancer

The training set **D** ∈ **ℝ** ^482 × 6539^ contains 482 cancer cell lines, 6538 genes, and drug IC_50_ values that correspond to a 482-dimensional column vector. The test set **T** ∈ **ℝ** ^24 × 6538^ consists of 24 breast cancer tumors and 6538 genes. The drug IC_50_ values for docetaxel (a chemotherapy drug) [46, 47] were downloaded from (http://genemed.uchicago.edu/~pgeeleher/cgpPrediction/). The cell line expression data were downloaded from the ArrayExpress repository [48] (accession number is E-MTAB-783, also available at https://www.ebi.ac.uk/arrayexpress/experiments/E-MTAB-783/?query=EMTAB783). The clinical trial data corresponding to the test set were downloaded from the Gene Expression Omnibus (GEO) repository (http://www.ncbi.nlm.nih.gov/geo/) with accession numbers GSE350 and GSE349 [49–51]. The data with accession numbers GSE350 and GSE349 contain 10 and 14 samples, respectively. If the remaining tumor was <25% or ≥25%, a breast cancer patient is considered to be sensitive or resistant, respectively, to docetaxel treatment. All the data were downloaded and processed according to the approach proposed by Geeleher et al. [11].

#### Data pertaining to multiple myeloma

The training set **D** ∈ **ℝ** ^280 × 9115^ contains 280 cancer cell lines, 9114 genes, and drug IC_50_ values that correspond to a 280-dimensional column vector. The test set **T** ∈ **ℝ** ^188 × 9114^ is composed of 188 multiple myeloma patients and 9114 genes. The drug IC_50_ values for bortezomib [52, 53] were downloaded from (http://genemed.uchicago.edu/~pgeeleher/cgpPrediction/), and the data for the cancer cell lines were downloaded from the ArrayExpress repository (accession number is E-MTAB-783 or available at https://www.ebi.ac.uk/arrayexpress/experiments/E-MTAB-783/?query=EMTAB783). The clinical trial data corresponding to the test set were downloaded from the Gene Expression Omnibus (GEO) repository (http://www.ncbi.nlm.nih.gov/geo/) with accession number GSE9782 [54]. The data were downloaded, processed and mapped according to Geeleher et al. [11].

#### Data pertaining to non-small cell lung cancer and triple-negative breast cancer

The training sets correspond to an 258 × 9508 matrix and an 497 × 9621 matrix for non-small cell lung cancer and triple-negative breast cancer, respectively. The test sets correspond to an 25 × 9507 matrix (excluding labels) and an 24 × 9620 matrix (excluding labels) for non-small cell lung cancer and triple-negative breast cancer, respectively. The data were downloaded from (http://genemed.uchicago.edu/~pgeeleher/cgpPrediction/) [11].

### Experimental methodology

Kernel-based methods, such as SVM and support vector regression (SVR), are popular machine learning algorithms and exhibit state-of-art performance in many applications [55, 56], including biological fields [57]. Therefore, in our experiments, we used SVR with linear kernel (SVR + L) and sigmoid kernel (SVR + S) as machine learning algorithms, coupled with our proposed link prediction algorithms (A1 or A2). We also employed our proposed link prediction algorithms with linear ridge regression (RR). In total, we considered 9 drug sensitivity prediction algorithms, as summarized in Table 1.

Each prediction algorithm was trained on the same training set, whose labels are continuous to yield models (see Methods section). Then, each model is applied to the same test set to yield predictions, as discussed in Methods section. The test set consists of the clinical trial expression data of patients, including baseline tumor expression data from primary tumor biopsies prior to treatment with an anticancer drug. The responses (i.e., labels) of the test set are categorical (e.g., either “sensitive” or “resistant”). These labels were clinically evaluated by the degree of reduction in tumor size to the given drug [11].

To evaluate whether the proposed approach exhibits stable superior performance as the sample size changes, we gradually reduced the sample size for the training set by 1 to 4% in each run. That is, we have 5 runs with sample sizes of 482, 478, 473, 468, and 463 and 280, 278, 275, 272, and 269 for the two datasets, respectively.

The accuracy of the prediction algorithms is measured using the *Area Under the ROC Curve* (AUC), as shown in [11]. The higher AUC an algorithm has, the better performance that algorithm achieves. We denote the mean of the AUC values averaged over the five runs of the test set as the MAUC. A run of the test set is defined as predictions of a learned model on the test set, such that the model is learned from the training set. The size of this training set is varied to assess the stability of prediction algorithms, in which a stable prediction algorithm is one for which the prediction accuracy on the test set does not change dramatically due to small changes in the size of the training set [58, 59]. This type of assessment is important in biological systems, in which the best prediction algorithm outperforms other algorithms many times in the conducted experiments. Statistical significance is measured between all pairs of the prediction algorithms.

The software employed in this study included support vector regressions with linear and sigmoid kernels in the LIBSVM package [60], ridge regression [11], gene selection using CUR and topLeverage functions in the rCUR package [45], and R code for processing the datasets and performance evaluation [11]. We used R to write the code for the link prediction algorithms and perform the experiments.

### Experimental results

Tables 2 and 3 show the AUC of 9 docetaxel and bortezomib, respectively, sensitivity prediction algorithms on clinical breast cancer or multiple myeloma trial data. For each variation in training set size the prediction algorithm with the best performance (i.e., the highest AUC) on the clinical trial data is shown in bold.

**Table 2:** AUC scores of docetaxel sensitivity prediction algorithms in breast cancer patients on the test set

m	482	478	473	468	463	MAUC
d	6538	6538	6538	6538	6538	−
A1 + SVR + L	0.878	0.864	**0.871**	0.857	0.871	0.868
A1 + SVR + S	0.871	0.857	0.814	0.828	**0.878**	0.849
A1 + RR	0.850	0.828	0.821	0.850	0.842	0.838
m + A1	246	244	242	239	237	−
d + A1	13,076	13,076	13,076	13,076	13,076	−
A2 + SVR + L	**0.892**	0.857	0.864	**0.864**	0.864	0.868
A2 + SVR + S	0.871	0.850	0.814	0.814	**0.878**	0.845
A2 + RR	0.857	0.842	0.835	0.835	0.835	0.841
m + A2	246	244	242	239	237	−
d + A2	13,000	13,000	13,000	13,000	13,000	−
B + SVR + L	0.835	0.814	0.800	0.821	0.835	0.821
B + SVR + S	0.842	**0.871**	0.864	0.857	0.857	0.858
B + RR	0.814	0.814	0.821	0.821	0.821	0.818

The algorithm with the highest AUC is shown in bold. MAUC = mean AUC

**Table 3:** AUC scores of bortezomib sensitivity prediction algorithms in multiple myeloma patients on the test set

m	280	278	275	272	269	MAUC
d	9114	9114	9114	9114	9114	−
A1 + SVR + L	0.668	0.669	0.665	0.663	0.656	0.664
A1 + SVR + S	0.638	0.623	0.637	0.642	0.662	0.640
A1 + RR	0.685	0.673	0.679	0.677	0.690	0.681
m + A1	145	144	143	141	140	−
d + A1	18,228	18,228	18,228	18,228	18,228	−
A2 + SVR + L	0.678	0.678	0.671	0.668	0.654	0.670
A2 + SVR + S	0.661	0.657	0.659	0.659	0.668	0.661
A2 + RR	**0.686**	**0.689**	**0.696**	**0.695**	**0.699**	0.693
m + A2	145	144	143	141	140	−
d + A2	9114	9114	9114	9114	9114	−
B + SVR + L	0.613	0.609	0.622	0.628	0.632	0.621
B + SVR + S	0.602	0.600	0.601	0.605	0.598	0.601
B + RR	0.614	0.611	0.603	0.607	0.606	0.608

The algorithm with the highest AUC is shown in bold. MAUC = mean AUC

Table 2 shows that our prediction algorithms perform better than the baseline prediction algorithms (i.e., B + SVR + L and B + SVR + S) including B + RR, which is a prediction algorithm proposed by Geeleher et al. Row “m” and “d”, shows the number of cell lines or genes, respectively, in the training set that were provided to each prediction algorithm. We provided the same training set to each prediction algorithm. Rows “m + A1” and “m + A2”, or “d + A1” and “d + A2” show the number of selected cell lines or genes, respectively, that were used in the prediction algorithms that employed our approach for learning the models. The results of our prediction algorithms are dominant compared with the baseline prediction algorithms that employ clinical trial data of breast cancer in terms of the AUC of four runs and the MAUC. In contrast to the baseline prediction algorithms, the performance of our prediction algorithms on the test set outperforms in terms of the AUC when we reduce the training set size.

Table 3 shows that our prediction algorithms perform better than the baseline prediction algorithms (i.e., B + SVR + L and B + SVR + S) and B + RR, which is a prediction algorithm proposed by Geeleher et al. Row “m” or “d”, respectively, shows the number of cell lines or genes, respectively, in the training set that were provided to each prediction algorithm. We provided the same training set to each prediction algorithm. Rows “m + A1” and “m + A2” or “d + A1” and “d + A2” show the number of selected cell lines or genes, respectively, used in the prediction algorithms that employ our approach for learning the models. The results of our prediction algorithms are dominant compared with the baseline prediction algorithms on the multiple myeloma clinical trial data in terms of the AUC of each run and the MAUC. In particular, A2 + RR achieves the highest mean AUC (MAUC) of 0.693 and performed the best in all runs. In contrast to the baseline prediction algorithms, the performance of A2 + RR on the test results in the best AUC as we reduce the training set size, which indicates that A2 + RR has a stable performance.

Table 4 shows the *p*-values of the two-tailed Wilcoxon signed rank test [61, 62] to measure the statistical significance between the prediction algorithms using clinical trial data of breast cancer and multiple myeloma patients. The *p*-values indicate that our A1 + SVR + L and A2 + SVR + L prediction algorithms significantly outperformed the baseline prediction algorithms B + SVR + L, B + SVR + S, and B + RR. The remaining prediction algorithms that employ our approach are not statistically different from B + SVR + S.

**Table 4:** *P*-values of Wilcoxon signed rank test (two-tailed) between all pairs of prediction algorithms

	A1 + SVR + S	A1 + RR	A2 + SVR + L	A2 + SVR + S	A2 + RR	B + SVR + L	B + SVR + S	B + RR
A1 + SVR + L	**0.0160**	0.5092	0.3077	0.0836	0.8807	**0.0051**	**0.0149**	**0.0051**
A1 + SVR + S	−	0.1675	**0.0208**	0.1282	0.0929	**0.0051**	0.1830	**0.0080**
A1 + RR	−	−	0.2846	0.5418	0.0672	**0.0051**	0.1388	**0.0076**
A2 + SVR + L	−	−	−	0.0587	0.5754	**0.0051**	**0.0207**	**0.0047**
A2 + SVR + S	−	−	−	−	0.1388	**0.0069**	0.0836	**0.0124**
A2 + RR	−	−	−	−	−	**0.0076**	0.1675	**0.0051**
B + SVR + L	−	−	−	−	−	−	0.5754	0.1609
B + SVR + S	−	−	−	−	−	−	−	0.2040

Values with statistical significance (*p* < 0.05) are shown in bold

**Fig. 3:**
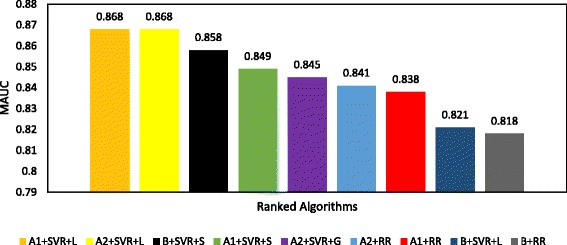
Mean AUC (MAUC) results of docetaxel sensitivity prediction algorithms in breast cancer patients ranked from the highest MAUC (*left*) to the lowest MAUC (*right*)

**Fig. 4:**
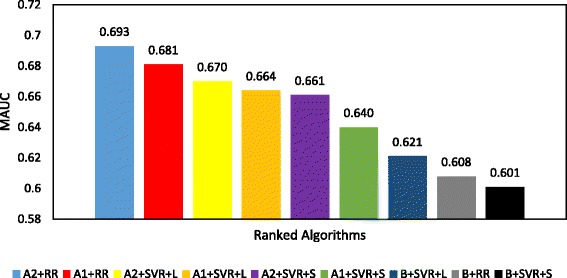
Mean AUC (MAUC) of bortezomib sensitivity prediction algorithms in multiple myeloma patients ranked from highest MAUC (*left*) to lowest MAUC (*right*)

Figures 3 and 4 show the ranking of all prediction algorithms from the highest to the lowest MAUC using clinical trial data pertaining to breast cancer and multiple myeloma patients, respectively. Each MAUC is calculated over the 5 runs of the clinical trial data. As shown in Figs. 3 and 4, our prediction algorithms outperform the baseline prediction algorithms [11] w.r.t the MAUC.

**Fig. 5:**
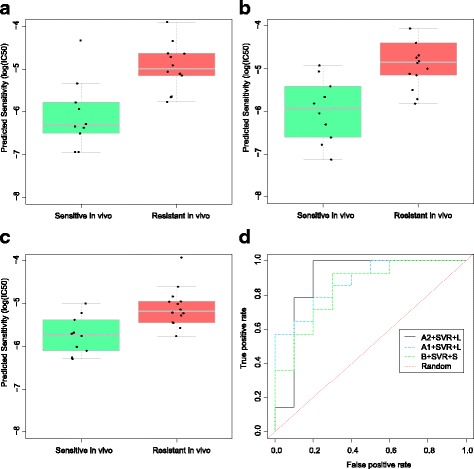
Prediction of docetaxel sensitivity in breast cancer patients. Strip charts and boxplots in (**a**), (**b**), and (**c**) show the differences in predicted drug sensitivity for individuals who are sensitive or resistant to docetaxel treatment using the prediction algorithms A2 + SVR + L, A1 + SVR + L and B + SVR + S, respectively, while (**d**) shows the ROC curves of prediction algorithms, revealing the proportion of true positives compared to the proportion of false positives. ROC = receiver operating characteristics

Figure 5 shows the predictions of three prediction algorithms on the test set (clinical data samples of 24 breast cancer patients) when the prediction algorithms were trained on a dataset with the size *m* = 482 (i.e., the complete training set without any reductions). Figure 5a–c show the predictions of A2 + SVR + L A1 + SVR + L and B + SVR + S, respectively. For A2 + SVR + L in Fig. 5a, the difference between the predicted drug sensitivity in breast cancer patients was highly statistically significant (*P*=472 × 10^−6^ from the result of a *t*-test) between the trial-defined sensitive and resistant groups. The result of A1 + SVR + L in Fig. 5b was also highly statistically significant (*P*=614 × 10^−6^ from a *t*-test). B + SVR + S in Fig. 5c achieved statistical significance (*P*=1176 × 10^−6^ from a *t*-test). Higher sensitivity or higher resistance, respectively, denote the greater or lesser effectiveness of the drug. In Fig. 5d, the ROC reveals AUC values of 0.892, 0.878 and 0.842 for A2 + SVR + L, A1 + SVR + L, and B + SVR + S, respectively, as shown in Table 2.

**Fig. 6:**
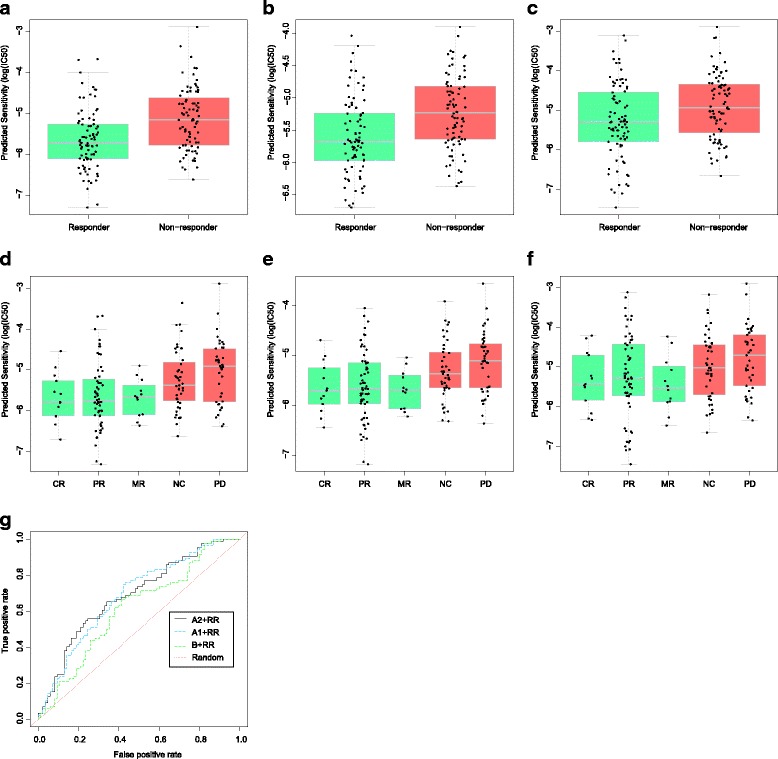
Prediction of bortezomib sensitivity in multiple myeloma patients. Strip charts and boxplots in (**a**), (**b**), and (**c**) show predicted drug sensitivity for in vivo responders and non-responders to bortezomib using A2 + RR, A1 + RR and B + RR prediction algorithms, respectively. Strip charts and boxplots (**d**), (**e**), and (**f**) further break down responders and non-responders of strip charts and boxplots (**a**, (**b**,) and (**c**) as showing CR, PR, MR, NC or PD using A2 + RR, A1 + RR and B + RR, respectively, prediction algorithms. (**g**) ROC curves illustrating estimated prediction accuracy of prediction algorithms. CR, complete response; PR, partial response; MR, minimal response; NC, no change; PD, progressive disease

In Fig. 6, the predictions of three prediction algorithms are reported on the test set (clinical trial data of 188 multiple myeloma samples of patients) when prediction algorithms learned models from a training set of size m = 280 (i.e., the training set without any reductions). Figure 6a–c show the predictions of the A2 + RR, A1 + RR, and B + RR, algorithms, respectively. For A2 + RR (Fig. 6a), the difference between the predicted drug sensitivity in multiple myeloma patients was highly significant (*P*=8 × 10^−6^ from a *t*-test) between trial-defined responder groups and non-responder groups. The result of A1 + RR was also highly significant (*P*=11 × 10^−6^ from a *t*-test), while B + RR achieved statistically significant result (*P*=2612 × 10^−6^ from a *t*-test). Figure 6d–f break down the responders and non-responders of Fig. 6a–c, respectively, to CR, PR, MR, NC or PD. In Fig. 6g, The ROC reveals AUCs of 0.686, 0.685, and 0.614 for A2 + RR, A1 + RR, and B + RR, respectively, as shown in Table 3.

We also evaluated the performance of prediction algorithms on the clinical trial data pertaining to non-small cell lung cancer patients and the triple-negative breast cancer patients. We observed similar results that our prediction algorithms noticeably outperform the baseline prediction algorithms (See Additional file 1: Tables S1 and S2).

It is worth mentioning that we also assessed the performance of other machine learning algorithms, including random forests [63], support vector regression with a polynomial kernel of degree 2, and support vector regression with a Gaussian kernel. Moreover, we applied other dimensionality reduction methods such as principal component analysis (PCA) [64] based on the prcomp package in R [65], sparse PCA [66, 67], non-negative and sparse cumulative PCA, and negative and sparse PCA [68, 69]. However, they did not exhibit acceptable predictive performance; consequently, their results are not included in this paper.

## Discussion

Gene (feature) selection is important to the success of the proposed method. After many years of biomedical research, some signaling pathways have been known for being implicated in various cancers. It is tempted to exploit this pathway information for feature selection. For example, we might consider adding the signaling pathways as a constraint to get reliable feature sets. Consequently, we assessed the performance of the proposed prediction algorithms using only the genes in the signaling pathways that are known to the cancers. We obtained inferior results (See Additional file 2 for details). It is noted that the current pathway information is limited. If we consider only those signaling genes, we may miss those important genes not identified yet by domain knowledge. This may hurt the overall performance as shown in our case. Therefore, a better strategy may be to include all genes but assign more weights to those signaling pathway genes. This is an interesting direction, and we leave it to our future work.

## Conclusion

In this paper, we introduce a link prediction approach to cancer drug sensitivity prediction. The benefit of introducing a link prediction approach is to obtain satisfactory feature representation for better prediction performance. We propose two algorithms that employ the link prediction approach: (1) A supervised link prediction algorithm, which selects better quality training cancer cell lines using a modified version of QBC; and (2) An extended supervised link prediction, which selects both better training cancer cell lines and a subset of important genes using state of the art CUR matrix decomposition.

In our study, the link prediction algorithms use two machine learning algorithms: support vector regression and ridge regression. The experimental results demonstrate the stability of the proposed link prediction algorithms, which outperform drug sensitivity prediction algorithms of an existing approach as measured by their higher and statistically significant AUC scores.

## Additional files

Additional file 1: Performance evaluation of prediction algorithms on clinical trial data pertaining to non-small cell lung cancer patients and triple-negative breast cancer patients. (DOCX 31 kb)

Additional file 2: Performance of prediction algorithms using signaling pathways as a constraint to get reliable feature set. (DOCX 25 kb)

## Abbreviations

A1: The supervised link prediction algorithm
A2: The extended supervised link prediction algorithm
AUC: Area under curve
B: The baseline approach
GEO: Gene expression omnibus
IC50: Half-maximal inhibitory concentration
MAUC: Mean area under curve
QBC: Query by committee
ROC: Receiver operating characteristic
RR: Ridge regression
SVR + L: Support vector regression with a linear kernel
SVR + S: Support vector regression with a sigmoid kernel
