# Supplementary material for: A link prediction approach to cancer drug sensitivity prediction
Source: BMC Syst Biol. 2017 Oct 3;11(Suppl 5):94. doi: 10.1186/s12918-017-0463-8 (PMC5629619; doi:10.1186/s12918-017-0463-8)
Supplement: Supplementary file 1 — Performance evaluation of prediction algorithms on clinical trial data pertaining to non-small cell lung cancer patients and triple-negative breast cancer patients. (DOCX 31 kb) [file 12918_2017_463_MOESM1_ESM.docx]

**Additional file 1**

Table S1 shows the performance of 9 erlotinib sensitivity prediction algorithms on non-small cell lung cancer data of patients. The prediction algorithms were evaluated using Spearman correlation test, where statistical significant results of prediction algorithms on the test set are shown in bold. The results indicate that prediction algorithms of the baseline were not statistically significant compared to ours. Specifically, A1+RR statistically significantly outperforming the baseline prediction algorithms, where *p* are calculated according to Spearman correlation test.

As the number of cell lines which respond to erlotinib is small and model is fitting a huge amount of noisy cell lines that affected the model, B+RR (proposed prediction algorithm via Geeleher et al.) results were not significant. Geeleher et al. [1] tackled this problem that caused poor performance as follows. They changed linear ridge regression and fitted model using logistic ridge regression model on the 15 most sensitive and 55 most resistant samples and then applied model to the test set (i.e., clinical trial data), where their model then achieved significant results (rho = 0.64 $p=5.3 \times10^{-4}$and from a Spearman’s correlation test). In contrast, our prediction algorithms achieved statistical significant results using linear ridge regression and this shows that our new feature representation is discriminative guiding the learning algorithm (RR) when incorporated with our prediction algorithms.

Table S1: Prediction of erlotinib sensitivity in NSCLC (non-small cell lung cancer) patients. Values with statistical significance (p<0.05) are shown in bold. Results are shown as (p/rho) according to Spearman correlation test

| m | 258 | 256 | 253 | 250 | 247 |
| --- | --- | --- | --- | --- | --- |
| d | 9507 | 9507 | 9507 | 9507 | 9507 |
| A1+SVR+L | **0.034**/-0.368 | 0.099/-0.265 | 0.111/-0.252 | **0.047**/-0.341 | **0.047**/-0.341 |
| A1+SVR+S | 0.053/-0.330 | 0.061/-0.315 | 0.078/-0.292 | **0.035**/-0.367 | 0.065/-0.310 |
| A1+RR | **0.007**/-0.480 | **0.018**/-0.421 | **0.013**/-0.441 | **0.011**/-0.453 | **0.010**/-0.458 |
| m+A1 | 134 | 133 | 132 | 130 | 129 |
| d+A1 | 19014 | 19014 | 19014 | 19014 | 19014 |
| A2+SVR+L | 0.394/-0.056 | 0.613/0.060 | 0.569/0.036 | 0.548/0.025 | 0.486/-0.007 |
| A2+SVR+S | 0.275/-0.125 | 0.382/-0.063 | 0.348/-0.082 | 0.290/-0.115 | 0.320/-0.098 |
| A2+RR | **0.028/-**0.385 | 0.127/-0.236 | 0.070/-0.302 | 0.069/-0.304 | 0.122/-0.241 |
| m+A2 | 134 | 133 | 132 | 130 | 129 |
| d+A2 | 9507 | 9507 | 9507 | 9507 | 9507 |
| B+SVR+L | 0.349/-0.081 | 0.399/-0.053 | 0.486/-0.007 | 0.426/-0.038 | 0.478/-0.011 |
| B+SVR+S | 0.571/0.037 | 0.668/0.091 | 0.610/0.059 | 0.584/0.045 | 0.599/0.053 |
| B+RR | 0.285/-0.119 | 0.350/-0.080 | 0.309/-0.104 | 0.266/-0.130 | 0.273/-0.126 |

Table S2 shows the performance of 3 cisplatin sensitivity prediction algorithms in triple-negative breast cancer patients’ data. The p-values used to evaluate prediction algorithms are from a linear regression model. Hence, we evaluate approaches that employ ridge regression. Geeleher et al. [1] assessed the response of 24 triple-negative breast cancer patients to neoadjuvant cisplatin therapy. Each patient is assigned to one of four drug response categories based on RECIST [2]. B+RR did not capture variability in clinical response (see Table S2 below)*­*. Our prediction algorithm achieved comparable results, which were not statistically significant.

Table S2: Prediction of cisplatin sensitivity in triple-negative breast cancer patients. Results are recorded according to p-values from a linear regression model

| m | 497 | 493 | 488 | 483 | 478 |
| --- | --- | --- | --- | --- | --- |
| d | 9620 | 9620 | 9620 | 9620 | 9620 |
| A1+RR | 0.1013 | 0.05781 | 0.08953 | 0.1775 | 0.1708 |
| m+A1 | 254 | 252 | 249 | 247 | 244 |
| d+A1 | 19240 | 19240 | 19240 | 19240 | 19240 |
| A2+RR | 0.192 | 0.1413 | 0.192 | 0.09515 | 0.1213 |
| m+A2 | 254 | 252 | 249 | 247 | 244 |
| d+A2 | 9620 | 9620 | 9620 | 9620 | 9620 |
| B+RR | 0.262 | 0.2055 | 0.185 | 0.2119 | 0.2306 |

1. Geeleher P, Cox NJ, Huang RS: **Clinical drug response can be predicted using baseline gene expression levels and in vitro drug sensitivity in cell lines**. *Genome Biology* 2014, **15**(3):R47-R47.
