# Supplementary material for: A link prediction approach to cancer drug sensitivity prediction
Source: BMC Syst Biol. 2017 Oct 3;11(Suppl 5):94. doi: 10.1186/s12918-017-0463-8 (PMC5629619; doi:10.1186/s12918-017-0463-8)
Supplement: Supplementary file 2 — Performance of prediction algorithms using signaling pathways as a constraint to get reliable feature set. (DOCX 25 kb) [file 12918_2017_463_MOESM2_ESM.docx]

**Additional file 2**

**Evaluating Signaling Pathways as a constraint to get reliable feature sets.**

We assessed the performance of prediction algorithms employing ridge regression including, the baseline prediction algorithm of Geeleher et al. (B+RR) on the test sets of breast cancer and multiple myeloma.

**For Breast Cancer**: We used the signaling pathways: Hedgehog (Hh), Notch, and Wnt, that have been reported as a novel therapeutic target in breast cancer [1-3]. These pathways consist of 217 unique genes and 113 of them could be found in our dataset which were used in our model. **For Multiple Myeloma (MM)**, We used Jak-STAT, PI3K-Akt, and NF-kappa B as the classic signaling pathways underlying MM [3, 4]. There are 512 unique genes in these pathways and 341 of them could be found in our dataset which were used in our model. We found that the performance of B+RR (i.e., the baseline) and A1+RR has significantly degraded where all achieved AUC below 0.60 on both test sets for breast cancer and multiple myeloma. This shows that removing discriminative features significantly degrade the performance of all prediction algorithms including the baseline (B+RR) as these discriminative features are crucial to improve the prediction performance.

1. Kamdje AHN, Etet PFS, Vecchio L, Muller JM, Krampera M, Lukong KE: **Signaling pathways in breast cancer: therapeutic targeting of the microenvironment**. *Cellular signalling* 2014, **26**(12):2843-2856.

2. Kubo M, Nakamura M, Tasaki A, Yamanaka N, Nakashima H, Nomura M, Kuroki S, Katano M: **Hedgehog signaling pathway is a new therapeutic target for patients with breast cancer**. *Cancer research* 2004, **64**(17):6071-6074.

3. Kanehisa M, Goto S: **KEGG: kyoto encyclopedia of genes and genomes**. *Nucleic acids research* 2000, **28**(1):27-30.

4. Chen L, Li Q, She T, Li H, Yue Y, Gao S, Yan T, Liu S, Ma J, Wang Y: **IRE1α-XBP1 signaling pathway, a potential therapeutic target in multiple myeloma**. *Leukemia Research* 2016, **49**:7-12.
